# Carbon composite micro- and nano-tubes-based electrodes for detection of nucleic acids

**DOI:** 10.1186/1556-276X-6-385

**Published:** 2011-05-16

**Authors:** Jan Prasek, Dalibor Huska, Ondrej Jasek, Lenka Zajickova, Libuse Trnkova, Vojtech Adam, Rene Kizek, Jaromir Hubalek

**Affiliations:** 1 Department of Microelectronics, Brno University of Technology, Technicka 10, CZ-61600 Brno, Czech Republic; 2 Department of Chemistry and Biochemistry, Mendel University in Brno, Zemedelska 1, CZ-61300 Brno, Czech Republic; 3 Department of Physical Electronics, Masaryk University, Kotlarska 2, CZ-61137 Brno, Czech Republic

## Abstract

The first aim of this study was to fabricate vertically aligned multiwalled carbon nanotubes (MWCNTs). MWCNTs were successfully prepared by using plasma enhanced chemical vapour deposition. Further, three carbon composite electrodes with different content of carbon particles with various shapes and sizes were prepared and tested on measuring of nucleic acids. The dependences of adenine peak height on the concentration of nucleic acid sample were measured. Carbon composite electrode prepared from a mixture of glassy and spherical carbon powder and MWCNTs had the highest sensitivity to nucleic acids. Other interesting result is the fact that we were able to distinguish signals for all bases using this electrode.

## Background

In the last two decades, nanomaterials in the form of nanotubes and nanowires have begun to be reported as promising materials for wide field of applications [1,2]. Such materials could be also used for fabrication of miniaturized electrodes. The nanostructured electrodes could be fabricated using several techniques. The easiest fabrication technique is to use a mixture of nanomaterial as filler with a suitable vehicle, which could be deposited on the electrode substrate using screen-printing, drop-coating, dip-coating, spraying, etc. [3,4]. The disadvantage of these nanocomposition-based electrodes is the irreproducible electrode surface with undefined active electrode area. The reproducible nanostructured electrode surface could be fabricated using lithography as a common tool for microelectronics devices implementation [5], anodization process for nanorods or nanotubes creation [6,7]. One of these techniques is the creation of vertically aligned multiwalled carbon nanotubes (MWCNTs) grown directly on the surface using chemical vapour deposition (CVD) [8]. The aim of this study was to fabricate MWCNTs and further to test the particles as a part of carbon composite electrodes with commercial carbon particles on detection of nucleic acids.

## Results and discussion

### Fabrication of vertically aligned MWCNTs

**Figure 1:**
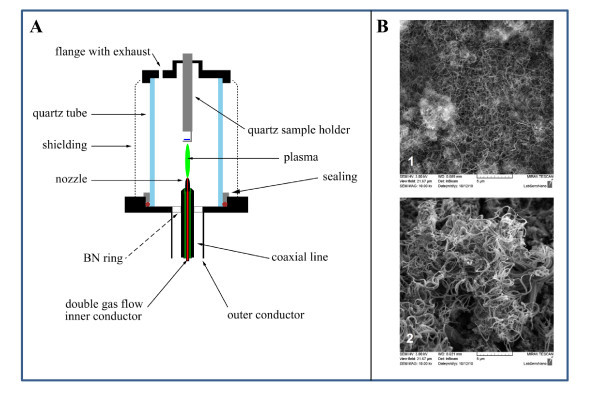
**Preparation and characterization of MWCNTs**. (**A**) Set-up of the apparatus for PECVD MWCNTs direct deposition (according to [8]). (**B**) Surface-enhanced micrographs of the electrodes fabricated on the Ag-based thick film paste (1) without and (2) with use of 10 nm Fe catalyst (Tescan, Czech Republic).

Primarily, vertically aligned MWCNTs were prepared. A detailed drawing of the current set-up of the apparatus for plasma enhanced CVD MWCNTs direct deposition is shown in Figure 1A. The apparatus consisted of a micro-wave generator, working at a frequency of 2.45 GHz, with a standard rectangular waveguide, transmitting the micro-wave power through a coaxial line to a hollow nozzle electrode. Ferrite circulator protected the generator from the reflected power by re-routing it to the water load. The coaxial line and the nozzle electrode accommodated a dual gas flow. The central conductor of the coaxial line was held in place by boron nitride ceramics. The outer conductor was terminated by a flange. A stub tuner was mounted to the waveguide for load matching, and the reactive mixture of CH_4_/H_2 _was added by a concentric opening instead of the set of holes in the outer housing. The plasma torch was enclosed by a quartz tube, 200 mm in length, with a duralumin shielding wrapped around the tube. The diameter of the quartz tube was 80 mm. The standard deposition mixture consisted of argon (700 sccm), methane (32 sccm) and hydrogen (255 sccm). Argon passed through the centre, whereas methane/hydrogen passed through the outer housing. The substrate for MWNT growth, a piece of alumina with sensor structure, was fixed on the quartz holder at the variable distance from the torch nozzle. It was heated by a heat exchanger with hot gas and surface recombination. The deposition temperature was 700°C. The deposition was done on the pure silver layer without any catalyst and on the 10-nm-thick Fe catalyst using the same underlay. The SEM comparison of the electrode materials fabricated on the Ag thick film paste with and without use of catalyst is shown in Figure 1B. It clearly follows from the results obtained that both pastes are covered with vertically aligned MWCNTs.

### Detection of nucleic acids

**Figure 2:**
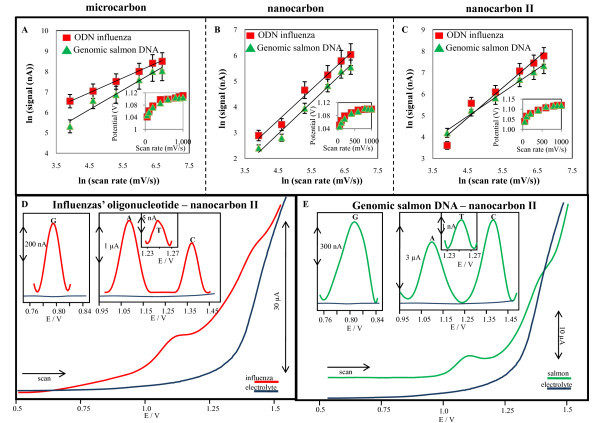
**Cyclic voltammetry**. The dependences of adenine peak height on *ln *of scan rates (50, 100, 200, 400, 600 and 800 mV/s) measured at (**A**) microcarbon, (**B**) nanocarbon and (**C**) nanocarbon II. In insets: dependencies of adenine peak potentials on *ln *of scan rate. Square wave voltammetry. SW voltammograms of (**D**) single strand oligonucleotide influenza (13 μg/mL), and (**E**) double strand genomic DNA (15 μg/mL). In insets: signals of all nucleic acid bases after baseline correction and smoothing of raw data.

Further, three carbon composite electrodes with different content of carbon particles with various shapes and sizes were prepared and tested on measuring of nucleic acids. The first carbon composite electrode (called as "microcarbon") was made of 90% "glassy carbon powder," where the particles are from glassy carbon material and they have spherical shape 2 μm (*w*/*w*, Sigma-Aldrich, USA) and 10% mineral oil (*m*/*w*, Sigma-Aldrich; free of DNase, RNase, and protease). The second one (called as "nanocarbon") was made of 60% "glassy carbon powder" (*w*/*w*, Sigma-Aldrich), 30% powdered cylinder carbon nanotubes (*w*/*w*, Sigma-Aldrich) and 10% mineral oil (*w*/*w*, Sigma-Aldrich). The third one was made of (called as "nanocarbon II") 60% "glassy carbon powder" (*w*/*w*, Sigma-Aldrich), 30% above prepared MWCNTs and 10% mineral oil. These prepared materials were housed in a teflon body having a 2.5-mm-diameter disk surface. Before measurements, the electrode surface was renewed by polishing with a soft filter paper in preparation for measurement [9-11], which was carried out in the presence of 0.2 M acetate buffer (5.0). The prepared carbon composite electrodes were used in the following experiments, in which genomic DNA isolated from salmon (genomic salmon DNA) and oligonucleotide single strand from influenza (ODN influenza; 5'-CAG TCG CAA GGA CTA ATC TGT TTG-3') were analysed. Carbon and/or graphite are of particular interest but its voltammetric response is complex as a result of its heterogeneous surface structure, where it exhibits both edge and basal plane sites and, depending upon how the graphite is aligned, the electrode may be predominantly basal or edge plane in character [12]. Numerous authors have been utilizing carbon nanotubes for the electro-oxidation of DNA [13]. The edge plane sites on graphite are generally accepted to exhibit far greater rates of electron transfer as compared to the basal plane sites. Further, the adsorption of species on the graphite surfaces also differs at the two sites [14]. Therefore, the versatility of the electrode was tested. Cyclic voltammetry was used for the detection of two nucleic acids' samples mentioned above and the basic electrochemical behaviour of nucleic acids at the surface of the above prepared carbon composite electrodes were studied. It is known that that cytosine, adenine, thymine and guanine give signals at carbon electrodes [15-17]. We found that both the nucleic acid samples gave all four signals corresponding to single bases at the tested electrodes. Adenine gave the highest signal; however, the sequence of height of the other bases measured on the electrodes differed. Guanine was the second-most electroactive bases at nanocarbon electrode followed by thymine and cytosine as well as at nanocarbon II electrode, but the height of cytosine was higher compared with thymine. At the surface of microcarbon electrode, the height of bases decreased in the following order thymine, guanine and cytosine. These changes can be associated with different surfaces and its affinity to single bases, which can subsequently influence the redox processes. To study the behaviour of bases on the surface of the electrodes, the dependences of adenine peak height on scan rate (50, 100, 200, 400, 600 and 800 mV/s) were determined. The logarithmic dependences are shown in Figure 2A-C for microcarbon, nanocarbon and nanocarbon II electrodes, respectively. It clearly follows from the results obtained that ODN influenza gave higher signal compared with genomic salmon DNA except ODN influenza signal measured under 50 mV/s at nanocarbon II. If we compared the sharpness of the dependencies, then the sharpest were those measured at nanocarbon II electrode followed by nanocarbon electrode and microcarbon electrode. Moreover, the dependencies of adenine peak potentials on scan rate were determined and are shown in insets in Figure 2A-C for microcarbon, nanocarbon and nanocarbon II electrodes, respectively (*R*^2 ^higher than 0.998). Based on both the logarithmic and linear dependencies, we found that the redox electrode process at all electrodes was diffusion-limited. Moreover, based on the Randles-Sevcik equation for a reversible and diffusion-controllable process, we estimated that the reaction exhibited nearly heterogeneous one-electron transfer. Moreover, the dependences of adenine peak height on concentration of nucleic acid sample were measured. The dependences were strictly linear for both nucleic acid samples with *R*^2 ^higher than 0.996. The slope of the obtained curves enhanced as follows: nanocarbon II > nanocarbon > microcarbon. It clearly follows from the results obtained that carbon composite electrode prepared from the mixture of glassy and spherical carbon powder and MWCNTs had the highest sensitivity to nucleic acids. Based on these results, nanocarbon II electrodes were further utilized for detection of both the nucleic acids' samples (25 μg/mL) using square wave voltammetry (not shown). We were interested in the issue whether we could detect signals of all bases due to such high sensitivity. Both the nucleic acids samples (15 μg/mL) were detected, and all purine and pyrimidine bases signals were observed (Figure 2D, E, for ODN influenza and genomic salmon DNA, respectively); peak potential about G = 0.8 V; A = 1.05, T = 1.25 and C = 1.35 V. Signals of the genomic DNA were higher (approx. 30%) in comparison with the oligonucleotide. Another interesting result is the fact that we were able to clearly distinguish signals for all bases by using baseline correction and smoothing (insets in Figure 2D, E).

## Conclusions

Based on these promising milestones of electroanalysis of nucleic acids together with the fact that electrochemistry is still one of the most sensitive analytical technique voltammetric methods can be considered as a suitable tool for detection of nucleic acids. We show the successful application of modern nano-technologies not only for detecting of nucleic acids but also for distinguishing of all bases signals.

## Methods

### Chemicals

All chemicals of ACS purity used and parafilm were purchased from Sigma-Aldrich Chemical Corp. (USA) unless noted otherwise. Salmon sperm DNA was bought from Applied Biosystems (USA). Synthetic oligonucleotides, which were purified using high performance liquid chromatography, were obtained from Sigma-Aldrich with following sequence: influenza HPI 5'-CAG TCG CAA GGA CTA ATC TGT TTG-3'. Stock standard solutions of the oligonucleotides (100 μg/mL) were prepared with water of ACS purity (Sigma-Aldrich) and stored in dark at -20°C. The concentrations of oligonucleotides and DNA were determined spectrophotometrically at 260 nm using spectrometer Specord 210 (Analytic Jena, Germany). Deionised water underwent demineralization by reverse osmosis using the instrument Aqua Osmotic 02 (Aqua Osmotic, Czech Republic), and then it was subsequently purified using Millipore RG (Millipore Corp., USA, 18 MΩ) - MiliQ water. The pH value was measured using inoLab controlled by the personal computer program (MultiLab Pilot; WTW, Germany).

### Electrochemical analysis

Electrochemical measurements were performed using AUTOLAB PGS30 Analyzer (EcoChemie, Netherlands) connected to VA-Stand 663 (Metrohm, Switzerland), using a standard cell with three electrodes. Carbon composite electrodes were employed as the working electrode. An Ag/AgCl/3 M KCl electrode served as the reference electrode. Glassy carbon electrode was used as the auxiliary electrode. For smoothing and baseline correction, the software GPES 4.9 supplied by EcoChemie was employed. Cyclic voltammetric parameters were as follows: potential step 5 mV; scan rates: 50, 100, 200, 400, 600 and 800 mV/s. Cyclic and square wave voltammetric measurements were carried out in the presence of acetate buffer pH 5. Square wave voltammetry parameters: potential step 5 mV, frequency 280 Hz. The samples measured by square wave voltammetry were deoxygenated before measurements by purging with argon (99.999%) saturated with water for 120 s. The temperature of supporting electrolyte was maintained by the flow electrochemical cell coupled with thermostat JULABO F12/ED (Labortechnik GmbH, Germany) and was 25°C [18].

## Abbreviations

CVD: chemical vapour deposition; MWCNTs: multiwalled carbon nanotubes.

## Competing interests

The authors declare that they have no competing interests.

## Authors' contributions

JP prepared the screen-printed alumina substrates for MWCNTs deposition, characterised MWCNTs using SEM and participated on paper drafting. DH carried out electrochemical measurements. OJ physically prepared MWCNTs. LZ participated on physically preparation of MWCNTs and on their characterization. LT treated electrochemical data and participated in preparation of the manuscript. VA participated in the design of the study and performed the analysis of the data. RK conceived of the study, and participated in its design. JH participated in design and coordination of the study and drafted manuscript.
